# Risk Stratification in Twin Pregnancies Complicated by GDM

**DOI:** 10.1155/2024/5203116

**Published:** 2024-09-21

**Authors:** Anja Catic, Florian Heinzl, Christian Göbl, Gülen Yerlikaya-Schatten, Theresa Reischer

**Affiliations:** ^1^ Department of Obstetrics and Gynecology Division of Feto-Maternal Medicine Medical University of Vienna 1090, Vienna, Austria; ^2^ Department of Obstetrics and Gynecology Division of Obstetrics Medical University of Graz 8010, Graz, Austria

**Keywords:** dietary management, GDM, gestational diabetes mellitus, insulin, pharmacotherapy, twin pregnancy

## Abstract

**Aims:** This study was aimed at assessing the association of oral glucose tolerance test (OGTT) glucose threshold levels and the requirement of insulin therapy in twin pregnancies with gestational diabetes mellitus (GDM).

**Methods:** In this post hoc analysis of a cohort study spanning 18 years, 446 patients with twin pregnancy and GDM (246 managed with lifestyle modification and 200 requiring pharmacotherapy) were included. We collected and evaluated maternal characteristics, as well as fasting, 1-h, and 2-h glucose concentrations from a standardized 75-g OGTT. The assessment methods included logistic regression analysis, positive and negative predictive values, area under the curve (AUC), and random forest analysis.

**Results:** The fasting (*p* < 0.01, OR: 1.03 [95% CI 1.01–1.05]) and 1-h (*p* < 0.01; OR: 1.01 [95% CI 1.00–1.02]) glucose levels during the OGTT were significantly associated with the subsequent need for insulin therapy, with thresholds of 95 mg/dL for fasting glucose and 184 mg/dL for the 1-h OGTT. Additionally, indications for insulin therapy were marked by thresholds of 108 mg/dL at G0, 215 mg/dL at G60, and 86 mg/dL at G120.

**Conclusion:** Identifying threshold values for insulin therapy and risk stratification in twin pregnancy are crucial for optimal patient management.

## 1. Introduction

Gestational diabetes mellitus (GDM) is well studied in singleton pregnancies. Glycemic characteristics of twin pregnancy with GDM are underrepresented in literature. The incidence rate of GDM in twin pregnancies has not uniformly been reported. Some studies have found twin pregnancies to be at higher risk for GDM [1–4], while others find similar rates of GDM [5, 6]. GDM is normally diagnosed by using a 2-h 75-g oral glucose tolerance test (OGTT). An OGTT can be performed already in the first trimester in high-risk pregnancies, but formal systematic evaluation for gestational diabetes occurs between 24 and 28 weeks of gestation as suggested by the International Association of Diabetes and Pregnancy Groups (IADPSG) criteria [7, 8].

Controlling blood glucose levels is the primary goal in managing women with GDM. An individual treatment plan consisting of lifestyle modification (dietary recommendations, exercise, and self-monitoring of blood glucose levels) and pharmacotherapy if indicated with insulin and/or metformin should be evaluated in each patient to ensure normal fetal development and better perinatal outcome [9]. Studies show different risk factors associated with the requirement for glucose-lowering medications for glycemic control in singleton pregnancies complicated by GDM such as age, prepregnancy BMI, prior history of GDM, glycated hemoglobin value at GDM diagnosis, and family history of Type 2 diabetes mellitus [10–14] as well as higher fasting and 2-h plasma glucose concentrations during OGTT [15, 16] to be strong indicators for insulin therapy (IT). But data on GDM in twin pregnancies is scarce. Previous studies have indicated that increased OGTT glucose concentrations were related to adverse gestational and fetal outcome [17, 18].

Women with hyperglycemia detected during pregnancy are at greater risk for perinatal morbidity and mortality, a higher risk for operative delivery, and at higher risk to develop Type 2 diabetes mellitus postnatally [19, 20] as well as cardiovascular disease [21]. Women with GDM and strict glycemic control have better pregnancy outcomes compared to females with GDM, who do not receive adequate treatment [9, 22]. Additionally, treatment of gestational diabetes reduces serious perinatal morbidity such as death, shoulder dystocia or even bone fracture, and nerve palsy [9].

After publishing our study on predicting clinical factors for pharmacotherapy in twin pregnancies complicated by GDM [23], we conducted a post hoc analysis to determine cutoff values for initiating IT and to assess risk stratification. This analysis was aimed at addressing the question of whether establishing cutoff values could minimize GDM-associated complications in twin pregnancies complicated by GDM.

## 2. Material and Methods

### 2.1. Design and Study Population

**Figure 1:**
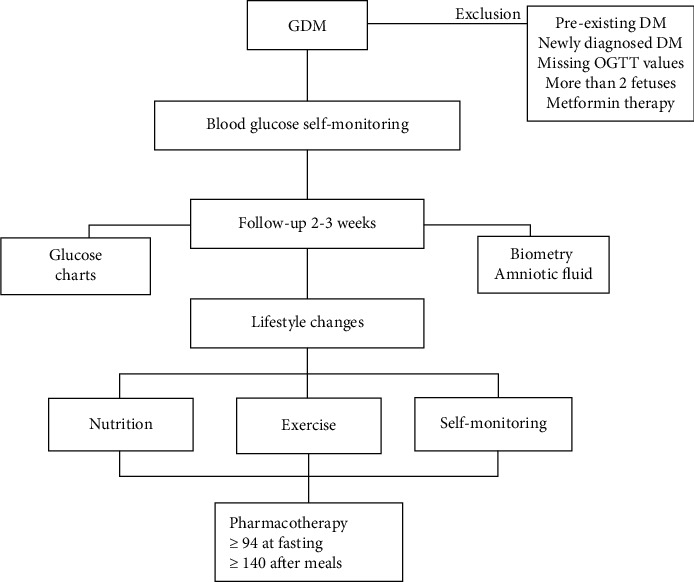
Study flowchart.

Detailed information about the study design has been reported previously [23]. Briefly, patients who were diagnosed with GDM (total 446 patients with GDM, 246 managed with lifestyle modification and 200 managed with IT) in twin pregnancy and seen at our outpatient clinic of the Medical University of Vienna, Division of Feto-Maternal Medicine, between January 2003 and December 2021 were retrospectively included in this post hoc cohort study. The diagnosis of GDM was determined using a standardized 75-g OGTT at 24–28 weeks of gestation [7, 8]. In cases with known risk factors, OGTT was performed earlier. The diagnostic thresholds for GDM were defined according to the IADPSG criteria after 2010 as follows: fasting blood glucose level92mg/dL, 1-h plasma glucose level180mg/dL, and 2-h plasma glucose level153mg/dL [7]. Before 2010, our cutoff values for the diagnosis of GDM were fasting blood glucose level95mg/dL, 1-h plasma glucose level180mg/dL, and 2-h plasma glucose level155mg/dL [8]. Patients were educated on blood glucose monitoring and given glycemic targets. Follow-up appointments occurred every 2–3 weeks, involving glucose level reviews and fetal growth assessments alongside amniotic fluid measurements. Lifestyle changes were the initial approach, including personalized plans for nutrition, exercise, and self-monitoring. Pharmacological intervention was considered if target glucose levels were not met (fasting94mg/dL or > 140 mg/dL after meals). Only patients with IT were included in the study. Patients managed with solely metformin or metformin + insulin have been excluded from the study population due to lacking guidelines in regard to metformin prescription. Data collected included age, pregnancy history, BMI, previous GDM, chorionicity, and OGTT results at fasting (G0), 60 min (G60), and 120 min (G120). The following data were excluded: preexisting diabetes, newly diagnosed diabetes, missing OGTT values, pharmacotherapy involving metformin, and pregnancies with more than two fetuses. A study flowchart is shown in Figure 1. Logistic regression as well as random forest analysis was performed for distinction of therapy (IT vs. lifestyle management). The area under the curve (AUC) was calculated for all three time points of the OGTT to predict the need for IT. Additionally, random forest was used for predicting the trimester in which IT is deemed necessary, as well as for a combination of these two situations.

The study obtained approval from the Ethics Committee of the Medical University of Vienna and was conducted in accordance with the principles outlined in the Declaration of Helsinki.

### 2.2. Outcome Variables

The central focus of this study revolved around two study questions: first and foremost, we aimed to determine if specific threshold values for risk stratification concerning IT in twin pregnancies complicated by GDM can be established. Secondly, we aimed to identify the key factors that play a role in distinguishing between the necessity for IT and lifestyle and dietary management (LSM), both overall and with respect to different pregnancy trimesters, as well as to uncover the factors that played a role in the decision to initiate IT during different stages of pregnancy.

## 3. Statistical Analysis

For logistic regression, we looked at two different thresholds (corresponding to 95% sensitivity and 95% specificity) in order to calculate positive predictive values (ppvs) as well as negative predictive values (npvs). On top, the AUC was calculated. For each forest, 500 trees were grown, minimal size of terminal nodes was set to 5, and the square root of all predictors present was used as the number of predictors sampled for the splits. The reduction in the mean Gini difference was used as the splitting rule. Out-of-bag errors as well as class prediction errors were calculated. Missing (numerical) values were imputed via chained equations using predictive values whereas patients with missing categorical data were discarded for this analysis.

## 4. Results

The study cohort included 446 pregnant mothers with twin pregnancies and GDM. Among them, 246 (55%) received lifestyle and dietary management (LSM), while 200 (45%) required treatment with insulin only. This contains the same study population as in [23]. More detailed information about patient characteristics and treatment arm is presented in [23] and Table 1. Briefly, the study population consisted of mothers with a median age of 32 years and a median BMI of 26 kg/m^2^. Most of the participants (373) did not have a history of GDM in a previous pregnancy, while 73 had prior GDM. Regarding OGTT results, the median fasting plasma glucose level was 92 mg/dL, the median 1-h OGTT was 182 mg/dL, and the median 2-h OGTT was 141 mg/dL. The study included a total of 309 pregnancies with dichorionic twins and 137 pregnancies with monochorionic twins. Mothers carrying a twin pregnancy and requiring pharmacotherapy (GDM-IT) were multipara and had a higher prepregnancy BMI, elevated plasma glucose concentrations during the fasting (95 mg/dL in the IT group vs. 90 mg/dL in the LSM group) and 1-h plasma glucose OGTT (184 mg/dL in the IT group vs. 180 mg/dL in the LSM group), and a higher rate of assisted reproduction. The methods of assisted reproductive technology (ART) are highlighted in Table S1, with the vast majority of our study population (82%) undergoing in vitro fertilization (IVF) treatment. However, no significant difference was found regarding the chorionicity of the pregnancy. In order to assess the factors leading to the need for pharmacotherapy, a logistic regression analysis was conducted using clinical parameters such as age, BMI, and various time points of the OGTT. The results showed significant associations between the need for pharmacotherapy and pregestational BMI, fasting glucose levels, and 60-min OGTT, with respective *p* values of < 0.0001 (OR: 1.07 [95% CI 1.03–1.12]), *p* ≤ 0.001 (OR: 1.03 [95% CI 1.01–1.05]), and *p* ≤ 0.01 (OR: 1.01 [95% CI 1.00–1.02]) (Table 1).

### 4.1. Primary Outcome

**Figure 2:**
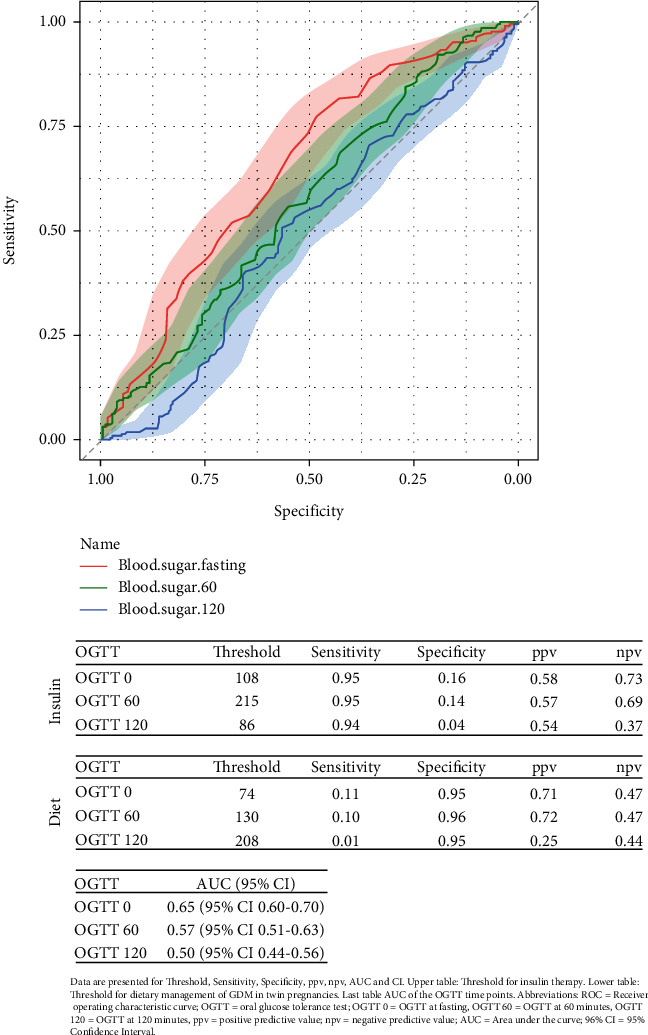
ROC curve of fasting OGTT, OGTT at 60 min, and OGTT at 120 min. Data are presented for threshold, sensitivity, specificity, ppv, npv, AUC, and CI. Top table: threshold for insulin therapy. Middle table: threshold for dietary management of GDM in twin pregnancies. Bottom table: AUC of the OGTT time points. Abbreviations: ROC = receiver operating characteristic curve; OGTT = oral glucose tolerance test; OGTT 0 = fasting OGTT; OGTT 60 = OGTT at 60 min; OGTT 120 = OGTT at 120 min; ppv = positive predictive value; npv = negative predictive value; AUC = area under the curve; 96% CI =95% confidence interval.

**Figure 3:**
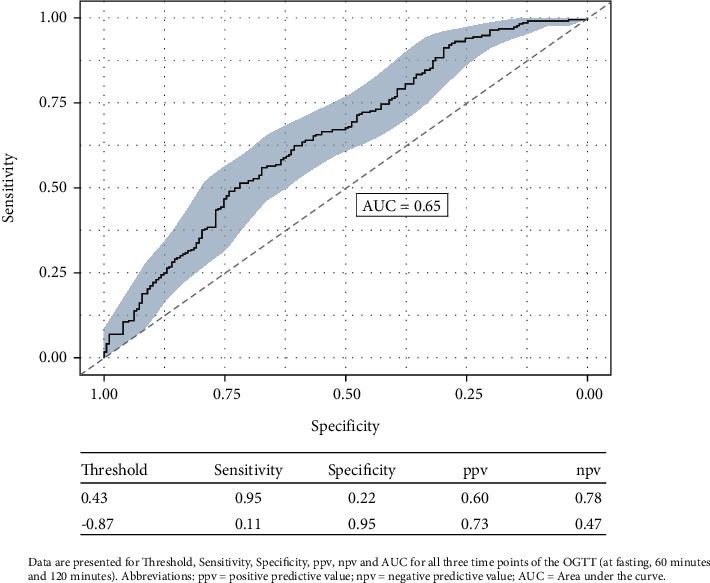
Multivariable logistic regression of fasting OGTT, OGTT at 60 min, and OGTT at 120 min. Data are presented for threshold, sensitivity, specificity, ppv, npv, and AUC for all the three time points of the OGTT (fasting, 60 min, and 120 min). Abbreviations: ppv = positive predictive value; npv = negative predictive value; AUC = area under the curve.

Receiver operating characteristic (ROC) curves were constructed to assess the ability of the OGTT at different time points to predict the need for IT. The cutoff level of 108 mg/dL at G0 was indicative of IT with a sensitivity of 0.95 and a specificity of 0.16 and a ppv of 0.58 and a npv of 0.73, followed by 215 mg/dL for the G60 with a sensitivity of 0.95 and a specificity of 0.14, as well as a ppv of 0.57 and a npv of 0.69. Further, the threshold of 86 mg/dL with a sensitivity of 0.94 and a specificity of 0.04 was calculated for the G120 with a ppv of 0.54 and a npv of 0.37, while results indicating dietary measures to be sufficient for the management of GDM in twin pregnancies have found the threshold for the G0 to be at 74 mg/dL with a specificity of 0.95 and a sensitivity of 0.11 and a ppv of 0.71 and a npv of 0.47. Further, for the G60, the threshold of 130 mg/dL with a specificity of 0.96 and a sensitivity of 0.10 was indicative of no requirement of insulin with a ppv of 0.72 and a npv of 0.47. For the G120, the cutoff was at 208 mg/dL with a specificity of 0.95 and a sensitivity of 0.01, as well as a ppv of 0.25 and a npv of 0.44. The results indicate that the fasting OGTT had the highest AUC value of 0.65 (95% CI 0.60–0.70), followed by 0.57 (95% CI 0.51–0.63) for the 60-min OGTT and 0.5 (95% CI 0.44–0.56) for the 120-min OGTT (Figure 2). The measurements for the G120 threshold seem to be instable and not reliable in this scenario and have not shown to be significant. While individual glucose measurements at G0 and G60 were significantly associated with the risk of requiring IT, their predictive performance was comparable when all three OGTT glucose values were combined using multivariable logistic regression (Figure 3).

### 4.2. Secondary Outcome

**Figure 4:**
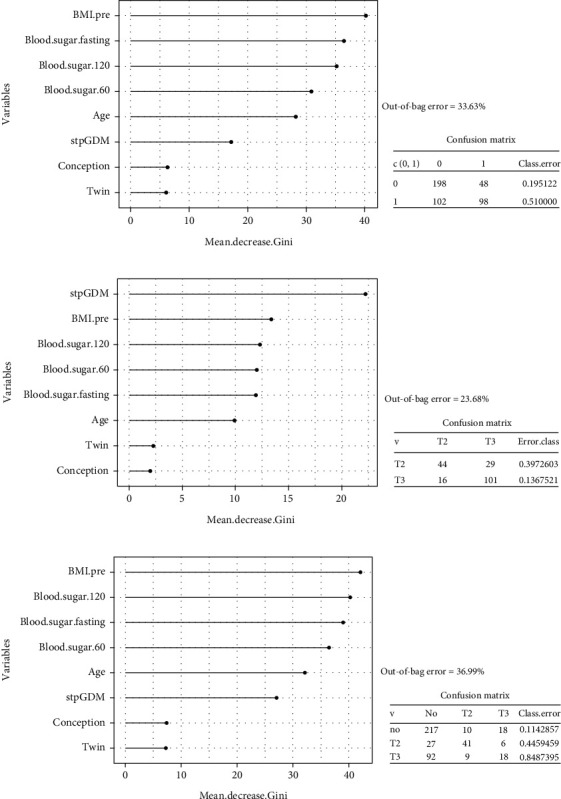
Random forest analysis.

In terms of the most influential variables, the Gini coefficients showed prepregnancy BMI as well as the time points of the OGTT to have the highest variable importance in discriminating between LSM and IT, while conception mode and chorionicity did not contribute substantially. With regard to the overall accuracy, we observed an out-of-bag error of 33.63%, whereas for the different prediction categories, we computed the following error rates: 48 and 96 (Figure 4(a)). When considering the Gini coefficients for IT, we found especially GDM in a previous pregnancy to be the most influential factor. But pregestational BMI, the OGTT at all time points, and maternal age also seem to be key factors in the prediction of initiation of insulin according to trimester. Conversely, factors such as conception mode and chorionicity did not contribute to the distinction, as determined by random forest analysis. In terms of overall accuracy, an out-of-bag error rate of 23.68% was observed. For the different prediction categories, error rates of 29 and 101 were calculated (Figure 4(b)). Prepregnancy BMI and OGTT measurements as well as age had the greatest impact in random forest analysis for comparison between LSM and IT according to trimester, while conception mode, chorionicity, or a previous history of GDM had minimal influence. The overall accuracy showed an out-of-bag error rate of 36.99%, with specific prediction categories having error rates of 18, 6, and 18 (Figure 4(c)).

## 5. Discussion

Stabilization of glucose levels plays a pivotal role in the management of GDM in twin pregnancies. Insulin is mainly used in pregnancy for the management of glucose levels; therefore, it is crucial to determine the threshold for insulin requirement during GDM in twin pregnancies in order to avoid adverse outcomes.

In this study, we aimed to investigate a risk stratification for the need of pharmaceutical intervention in twin pregnancies complicated by GDM.

We could previously demonstrate that higher fasting glucose levels and elevated 1-h glucose levels during the OGTT were associated with a greater likelihood of requiring IT in twin pregnancies with GDM. These findings suggest that fasting and 1-h glucose levels can serve as independent predictors for the need of insulin in GDM in twin pregnancies. Notably, we further identified threshold levels of 108 mg/dL for fasting glucose and 215 mg/dL for 1-h OGTT glucose that were indicative of requiring pharmacotherapy. For the 2-h OGTT, a threshold of 86 mg/dL was already indicative of a requirement of pharmacotherapy with insulin, while thresholds of 74 mg/dL for the fasting glucose levels and 130 mg/dL for the 1-h OGTT and 208 mg/dL for the 2-h OGTT level showed promising cutoff values for dietary measures. When evaluating all the three time points of the OGTT, blood sugar levels at fasting and 60 min exhibited robust predictive capabilities for the necessity of pharmaceutical intervention. Previous research has investigated risk factors and thresholds for initiating IT in GDM, but these studies have primarily focused on singleton pregnancies [16, 24, 25]. To the best of our knowledge, there is a lack of comparable studies specifically examining GDM twin pregnancies. Therefore, we can only extrapolate conclusions from the existing literature published on singleton pregnancies which thus far found that a fasting glucose level of 102.4 mg/dL and a 2-h glucose level of 194.4 mg/dL during the OGTT were predictive factors for the use of insulin in women diagnosed with GDM. These thresholds indicated a higher likelihood of requiring IT in the management of GDM based on the study's findings [16]. Another study reported a specific threshold level of ≥ 89.5 mg/dL in fasting glucose during the OGTT as a distinguishing factor for cases that required insulin in addition to nutritional therapy [24]. All measurements in singleton pregnancies show lower cutoff levels when compared to our study's calculations in twin pregnancies. Further, in a retrospective study involving 435 women with gestational diabetes, it was observed that fasting plasma glucose was not highly sensitive as a screening tool. However, it was found that a fasting plasma glucose level of 86 mg/dL could predict the need for insulin treatment with a sensitivity of 90% [25]. We showed a sensitivity of 95% for the threshold of 108 mg/dL in fasting plasma glucose.

Other studies have indicated that pregnant women with a fasting glucose level of 95 mg/dL or lower may require approximately 2 weeks of dietary therapy to achieve good glycemic control. These findings suggest that strict adherence to dietary modifications can effectively manage glucose levels in gestational diabetes [26]. Our study found a fasting glucose level of 74 mg/dL to be indicative of dietary measures for optimal glucose control in twin pregnancies. Langer has demonstrated a robust positive correlation between fasting glucose levels exceeding 105 mg/dL and the occurrence of maternal and perinatal complications in gestational diabetes. Moreover, this threshold value has been identified as an indicator for initiating IT [27]. This was supported by Akinci et al. [28]. The studies conducted in singleton pregnancies align with our own findings in twin pregnancy and support the notion that fasting glucose levels are a strong predictor of the need for antenatal insulin treatment in cases of gestational diabetes. However, in twin pregnancies, our study found that both the 1-h and fasting OGTT levels were relevant in predicting the requirement for insulin treatment [23] and showed cutoff values of 108 mg/dL for fasting and 215 mg/dL for the 1 h OGTT to be relevant for the initiation of IT, while the measurements for the 2 h OGTT seem to be unstable and not very promising in GDM twin pregnancies. A recent study compared GDM in twin pregnancy versus singleton pregnancy and found that GDM twin pregnancy had a higher incidence of abnormal 2-h OGTT values [29].

Further, in regard to our second study question, we could clearly demonstrate that prepregnancy BMI and all time points of the OGTT emerged as pivotal factors in distinguishing between those requiring LSM and those necessitating IT. These factors maintained their significant impact in forecasting the initiation of IT during distinct trimesters. Moreover, a prior history of GDM emerged as a strong and reliable indicator for predicting the need for IT, particularly when categorized by trimester. These have also been classified as predictive clinical factors for the initiation of IT and have been discussed previously [23]. Additionally, maternal age ≥ 35 years, overweight or obesity, and chronic hypertension have been identified as significant risk factors for GDM during twin pregnancies, adding that GDM-IT during twin pregnancies may have a higher risk of preterm birth and extrauterine growth restriction [30].

In our study population, the vast majority of patients underwent IVF treatment in the assisted reproduction category, yet ART did not perform well in the models differentiating between IT and LSM. Factors such as advanced maternal age, nulliparity, higher prepregnancy BMI, and conception by ART have been identified as risk factors for the development of GDM in women during twin pregnancies [31, 32].

It is worth noting that the variations in cutoff values between studies could be attributed to factors such as the number of fetuses (singleton vs. twins), differences in study methodologies, varying target glucose levels, or regional differences in clinical practices. While our AUC for the ROC curve of OGTT measurements may not have achieved optimal performance, our findings provide valuable insights into the appropriate thresholds and direction for initiating IT in twin pregnancies complicated by GDM. Given the lack of a universal consensus regarding the timing of initiation of IT in GDM during pregnancy, our study offers a potential framework for a personalized risk stratification approach. By assessing individual patient OGTT results, our research could help identify those who may require IT to effectively manage plasma glucose levels and, in turn, minimize adverse pregnancy outcomes.

With all this knowledge, it is crucial to inform patients about the increased likelihood of experiencing a recurrence of GDM in future pregnancies and a higher risk for Type 2 diabetes mellitus and cardiovascular disease [33]. Additionally, twin pregnancy was strongly associated with all adverse perinatal outcomes except macrosomia and increases the risk for neonatal hypoglycemia [34]. It has been recently reported that GDM seems to be protective for the occurrence of SGA neonates in twin pregnancies [35]. Early identification of risk factors allows for appropriate monitoring and intervention strategies to be implemented promptly. Individual patient characteristics and clinical judgment should always be considered when making treatment decisions for GDM.

### 5.1. Strengths and Limitations

Strengths of our study include a large study population that underwent regular and closely monitored follow-ups, including routine ultrasound scans. Secondly, we were able to leverage comprehensive documentation of the study participants, resulting in a substantial dataset. Regarding the changes in the cutoff values for diagnosing GDM, it is worth noting that these adjustments do not impact our calculations nor the outcome values. Therefore, these changes are not significant for our study but were mentioned to provide a comprehensive perspective.

Our study has some limitations, primarily due to its retrospective nature. Unmeasured fetal or placental factors that can influence insulin resistance might affect antenatal insulin treatment outcomes. Additionally, our data lacks information on the ethnicity of our study population. The absence of data on ethnicity may lead to an incomplete understanding of how certain ethnic groups might have a higher risk of developing metabolic diseases. Furthermore, there were missing variables that needed to be calculated to obtain results. Even though cutoff values have been established in single-center studies, there is still lacking evidence for external validation for the results obtained in single centers.

### 5.2. Conclusion

OGTT threshold levels of 108 mg/dL for fasting glucose and 215 mg/dL for 1-h OGTT are good predictors for the initiation of IT in twin pregnancies complicated by GDM.

## Figures and Tables

**Table 1:** Maternal characteristics of the study population. Logistic regression for the initiation of insulin therapy versus lifestyle modification in twin pregnancies with GDM.

	**Total**	**Age**	**BMI**	**Parity**	**G0**	**G60**	**G120**	**pGDM**	**hpGDM**	**npGDM**	**Conception mode**	**Assisted reproduction**	**Spontaneous pregnancy**	**Chorionicity**	**DC**	**MC**
*n*	446	32 (29–36)	26 (22–30)	1 (1–3)	92 (83–98)	182 (157–196)	141 (120–162)		73	373		152	294		309	137
GDM-IT	200	33 (30–36)	27 (23–32)	2 (1–3)	95 (88–102)	184 (161–203)	139 (118–165)		59	148		81	126		146	61
GDM-LSM	246	32 (29–36)	25 (22–28)	1 (1–2)	90 (82–95)	180 (156–192)	143 (122–160)		14	225		71	168		163	76
*p* value		> 0.05	< 0.01		< 0.01	< 0.05	> 0.05	< 0.01			< 0.05			> 0.05		
OR			1.07		1.03	1.01										
95% CI			1.03–1.12		1.01–1.05	1.00–1.02										

*Note:* Data are median and interquartile range (IQR) for pregnant women affected by GDM and treated with lifestyle modification or requiring insulin therapy. Age (years).

Abbreviations: 95% CI: 95% confidence interval; BMI: pregestational body mass index (kg/m^2^); DC: dichorionic twins; G0: fasting plasma glucose (mg/dL); G120: plasma glucose 120 min after oral glucose load (mg/dL); G60: plasma glucose 60 min after oral glucose load (mg/dL); GDM-IT: pregnant women affected by GDM and requiring insulin therapy; GDM-LSM: pregnant women affected by GDM and treated with lifestyle modification; hpGDM: history of previous pregnancy with gestational diabetes mellitus; MC: monochorionic twins; npGDM: no previous pregnancy with gestational diabetes mellitus; OR: odds ratio; pGDM: previous pregnancy with gestational diabetes mellitus.

## Data Availability

The data presented can be obtained by contacting the corresponding author. However, due to privacy concerns, the data are not publicly accessible.
