# Supplementary material for: Risk Stratification in Twin Pregnancies Complicated by GDM
Source: J Diabetes Res. 2024 Sep 21;2024:5203116. doi: 10.1155/2024/5203116 (PMC11438509; doi:10.1155/2024/5203116)
Supplement: Supporting Information — Additional supporting information can be found online in the Supporting Information section. Table S1. Type of reproductive methods. [file 5203116.f1.docx]

Supplementary Table 1. Type of reproductive methods

| ART | GDM - IT | GDM - LSM | total |
| --- | --- | --- | --- |
| Stimulation | 2 (100%) | 0 | 2 |
| ICSI | 13 (59%) | 9 (41%) | 22 |
| Heterologous insemination | 1 (50%) | 1 (50%) | 2 |
| IVF | 64 (52%) | 60 (48) | 124 |
| Egg donation | 1 (50%) | 1 (50%) | 2 |
|  | 81 (53%) | 71 (47%) | 152 |

Note: Data are Number n (%) for assisted reproduction methods; Abbreviations: ART: assisted reproductive technology; GDM – IT: gestational diabetes mellitus treated with Insulin; GDM-LSM: gestational diabetes mellitus treated with lifestyle modification; ICSI: IntraCytoplasmic Sperm Injection; IVF: InVitro Fertilisation
